# A Review of Brucellosis in Iran: Epidemiology, Risk Factors, Diagnosis, Control, and Prevention

**DOI:** 10.18869/acadpub.ibj.21.6.349

**Published:** 2017-11

**Authors:** Maryam Golshani, Saeid Buozari

**Affiliations:** Department of Molecular Biology, Pasteur Institute of Iran, Tehran, Iran

**Keywords:** Brucellosis, Iran, Epidemiology, Transmission, Subunit vaccine

## Abstract

Brucellosis caused by species of *Brucella* is among the most prevalent zoonoses with the annual incidence of half a million cases globally. Most parts of Iran are endemic for brucellosis, and the annual incidence of the human and animal brucellosis is still high. At present, there is no safe and protective human vaccine against brucellosis, and the only preventive strategy is animal vaccination, which harbors significant disadvantages. Considering the identification of many immunogenic proteins in *Brucella*, several studies have recently been performed to evaluate the vaccine potency of such antigens as a new subunit vaccine candidate. This review represents an overview of brucellosis in Iran, including epidemiology, transmission routs, diagnosis, and treatment. Moreover, it mainly highlights the history of brucellosis control and prevention in Iran, including eradication programs, vast livestock vaccination programs, and subunit vaccine studies. It also discusses major problems that the country encounters with disease control. In recent years, Persian scientists have focused on evaluating the efficacy of best *Brucella* immunogens *in vivo* to introduce a new subunit vaccine. The results of some studies could demonstrate the vaccine potential of some immunogens.

## INTRODUCTION

Brucellosis is one of the most prevalent bacterial zoonotic diseases causing significant economic losses due to the livestock abortion, and also it is possibly a life-threatening multi-system disease in human[1,2]. In 1886, *Brucella melitensis* was first isolated by David Bruce from the spleen of a British soldier who had died of a febrile illness, which was known as Malta fever and it was common among military personnel stationed on the island of Malta. The bacterium was named *Micrococcus melitensis*, with ‘melitensis’ derived from the Roman name for Malta, ‘Melita’. In 1897, *Bacillus abortus* was identified as the cause of contagious abortion in cattle by Bernhard Bang. Later, in 1917, it was found that the causes of the two diseases were identical, and renamed *Brucella* in honor of Bruce[3,4]. In Iran, *B. melitensis* was primarily isolated from the human blood culture in 1932, and in the cattle population, *B. abortus* was isolated from an aborted fetus in 1944[5].

### Etiology

*Brucella* are small (0.5-0.7 by 0.6-1.5 µm), Gram-negative, intracellular, nonmotile, nonsporulating, nontoxigenic, nonfermenting, facultative cocco-bacilli[6,7]. At present, based on host preferences and phenotypic differences, the genus of *Brucella* is classified into over ten species[1,8,9]. Recently, a novel species, *B. inopinata* (strain BO1), which is associated with a breast implant infection in a patient in Oregon, was isolated from a wild rodent in Australia[9]. Table 1 classifies *Brucella* species based on their virulence, host preference, and biovars[1,5,8-18].

**Table 1:** Classification of *Brucella* spp. Pathogens

*Brucella* species	Host preferences	Pathogenic potential	Number of biovars	Biovars isolated in Iran[5,13-18]	Endemic biovars in Iran[5]	Most prevalent biovars[5]	Hosts
*B. melitensis*	Sheep, goats, and cattle	high	3	1, 2, 3	1	1	Sheep, goat, camel, dog and cattle
							
*B. abortus*	Cattle	moderate	7	1, 2, 3, 4, 5, 6, 9	3	3, 1, 5	Cattle, horse, sheep and goat
*B. suis*	Pigs, reindeer, and hares	moderate	5	1, 3	-	-	Pig
*B. ovis*	Sheep	low	1	-	-	-	-
*B. canis*	Dogs	-	1	-	-	-	-
*B. neotomae*	Desert wood rats	-	1	-	-	-	-
*B. pinnipedialis*	Seals	low	-	-	-	-	-
*B. microti*	Common vales	-	-	-	-	-	-
*B. ceti*	Cetaceans	low	-	-	-	-	-
*B. inopinata*	Wild rodent in Australia	low		-	-	-	-

### Epidemiology

Annually, more than 500,000 new human cases of brucellosis are reported worldwide[1,19]. The prevalence rates of brucellosis are more than 10 cases per 100,000 population in some countries. However, it is believed that the incidence of disease is underestimated since for each reported case, 26 cases are not detected The incidence rate in endemic areas is under 0.03 to more than 200 in 100,000 persons[20,21]. Many parts of the world are still endemic for brucellosis, including the Middle East (Iran), Africa, Latin America, Central Asia, and the Mediterranean Basin. Most parts of Iran are endemic for the disease, especially the areas where human lives in a close contact with livestock[1,22,23]. According to the report of the Ministry of Health and Medical Education (as cited in Zeinali *et al*.’s[24] and Esmaeili’s[25] works), based on the incidence of human brucellosis in Iran, provinces are categorized into four types (Table 2). Moreover, in a study on trends of human brucellosis between 1991 and 2008, the mean annual incidence of human brucellosis was reported as 43.24 per 100,000 population[26]. In a recent study by Rostami *et al*.[27] on 1698 patients from 30 provinces of Iran, the mean of brucellosis incidence was reported to be 29.83 in 100,000 population (55% males and 45% females). In another study, Kassiri *et al*.[28] indicated that the incidence of Brucellosis in West of Iran was 59.31 per 100,000 population (34.9% females and 65.1% males), and nearly 95.2% of human cases were living in rural and 4.8% in urban areas. Moreover, the incidence rate of brucellosis in children in this region of Iran was 41.4 per 100,000 population (68.9% boys and 31.1% girls), among which 87.8% resided in rural areas[29]. On the other hand, Bokaie *et al*.’s[30] report showed the incidence of brucellosis as 37 per 100,000 population in East of Iran. Regarding the prevalence of brucellosis by age, the median age of 31.3 years has been reported by Zeinalian Dastjerdi *et al*.[31]. The prevalence of brucellosis in men is higher than in women in industrialized countries; however, in Iran, due to the close cooperation of women with men in ranching and farming occupations, the disease is also highly frequent in women[32]. In Sofian’s work[33], 55.3% of the affected pateints were male and 44.7% were female (with the mean age of 33.37±21.3). Moreover, the global investigation of the seasonal pattern of brucellosis shows that the disease is more prevalent in the first half of the year, which is the livestock’s offspring season [34]. The rate of brucellosis enhances during spring and summer due to factors such as direct contacts between ranchers and aborted fetuses as well as consumption of contaminated dairy products. In contrast, the rate of the disease decreases in the second half of the year. Determining seasonal pattern of Brucellosis in Iran using meta-analysis showed that the highest incidence of brucellosis occurred during spring and summer, while the lowest incidence occurred during winter and autumn[34]. Rostami *et al*.[27] also indicated that the highest and lowest cases of brucellosis are observed in spring and autumn, respectively.

**Table 2:** Classification of Iran’s provinces based on human brucellosis incidence[24,25]

Type of incidence	Provinces	Incidence of *Brucella* per 100,000 population
Very high	East Azerbaijan, Hamadan, Markazi, Lorestan, Kermanshah, West Azerbaijan, and South Khorasan	31-41
High	Kordistan, Razavi Khorasan, and Zanjan	21-30
Moderate	Golestan, Ilam, Qazvin, Semnan, Chaharmahal and Bakhtiari, Ardabil, Kerman, Mazandaran, Yazd, North Khorasan, and Fars	11-20
Low	Bushehr, Khuzestan, Kohgiluyeh and Boyer-Ahmad, Alborz, Tehran, Gilan, Hormozgan, Sistan and Baluchistan, and Qom	0-10

### Transmission and risk factors

The prevalence of human brucellosis is dependent upon factors such as husbandry practices, dietary habits, methods of processing milk, and dairy products, as well as environmental sanitation[10]. Brucellosis is transmitted from infected livestock to human via ingestion (unpasteurized milk or dairy products), inhalation, conjunctiva, or skin abrasions[22,35]. Brucellosis is usually considered as an occupational disease because it occurs mostly in abattoir workers, veterinarians, lab technicians, hunters, farmers, and livestock producers. The transmission of brucellosis is not usually from person to person; nevertheless, it may be transmitted via blood transfusion, bone marrow transplantation, sexual contact, or congenital[10,19,22]. Identifying the major risk factors for brucellosis is very important to reach a comprehensive understanding of the nature of the disease and its transmission routes for eradication of human brucellosis[33]. In Iran, the main risk factors for brucellosis are consumption of unpasteurized dairy products (especially raw milk and fresh cheese), direct contact with animals and animal husbandry, laboratory and veterinary professions, and the presence of another case of brucellosis at home[33,36-39]. Furthermore, the geographic situation of Iran is considered as an important risk factor for the extension of contagious diseases, particularly from the Eastern and Western neighbor countries such as Iraq, Pakistan, and Afghanistan. Due to the lack of accurate control programs for animal diseases in these countries, brucellosis is endemic in these areas and therefore, there is a risk of brucellosis transmission from these countries to Iran[25].

### Clinical manifestations

Based on the course of the disease, human brucellosis is classified into three forms: (1) acute brucellosis characterized by weakness, undulant fever, headaches, myalgia, fine red rash, splenomegaly, hepatomegaly, and gastrointestinal symptoms. The acute phase may end in death, curing, transition into a sub-acute or chronic form; (2) sub-acute brucellosis characterized by almost all symptoms typical of the acute course but milder; (3) chronic brucellosis in which long-term signs and symptoms may include fatigue, recurrent fevers, arthritis, endocarditis, and spondylitis[7,22,41,42]. In a study performed by Beheshti *et al*.[37], sweating, myalgia, and weakness were symptoms that were highly predictive for having a positive serological test result. Moreover, the prevalence of arthritis in children has been reported to be 24 out of 96 cases (25%) diagnosed with brucellosis[43]. The most common manifestations were fever (87.5%) and fatigue (75%). Three cases of *Brucella* infection of the thyroid gland were reported by Azizi and Katchoui[44]. All three cases were female, and two were from rural areas in Tehran Province.

### Diagnosis

Microscopic examination of stained smears is a useful tool for probable diagnosis, especially if it is confirmed by other tests. *Brucella* are arranged as a single coccobacilli or short rods, though they are sometimes in pairs or small groups[23]. Although blood culture is known as the gold standard in the diagnosis of brucellosis, it has major limitations such as being time-consuming and needing biosafety level 3 and expert personnels[45]. Culture or serology is the definitive diagnostic methods for human brucellosis. Blood, tissue samples, pus and cerebrospinal, joint, ascitic, or pleural fluid can be used for the isolation of *Brucella*[10,46]. Automated culture systems, replacing the traditional biphasic Ruiz-Castaneda system, are now a safer and faster method of diagnosis[47,48]. According to the study performed by Purcell *et al*.[7] the detection rate of BACTEC (Becton Dickinson Diagnostic Instrument Systems, Sparks, MD, USA), Myco/F Lytic medium in conjunction with BACTEC 9240 blood culture system was 80%. However, the detection rate of the pediatric Peds Plus/F or adult Plus Aerobic/F medium in conjunction with BACTEC 9240 blood culture system was 100%[7]. The sensitivity of both traditional and automated methods for the acute form had been reported to be about 90%. However, the sensitivity of the Biphasic Ruiz-Castaneda system in the chronic form is less than 20% in comparison to the sensitivity of 70% for the automated systems[49,50]. Traditionally, alternative brucellosis diagnostic methods in the absence of culture facilities are serological tests such as the Rose Bengal test, the serum agglutination test (SAT), and the antiglobulin or Coombs’ test, which are based on the reactivity of antibodies against smooth lipopolysaccharide (LPS). Usually, the Rose Bengal test is used as a screening test, and positive samples are confirmed by the SAT. The sensitivity of the Rose Bengal plate test is more than 99%, and the rate of false-negative results is infrequent[7,10,19,48]. Enzyme-linked immune-sorbent assay (ELISA) that measures IgG, IgM, and IgA antibodies has advantages of having high sensitivity and possibility of better interpretation of the clinical situation. However, the specificity of ELISA is less in comparison to the agglutination tests[19,48]. Finally, molecular detection methods are rapid and convenient for the diagnosis of human brucellosis and may improve sensitivity relative to the culture. *Brucella* is detectable from serum, blood, pus, and tissue, but the usage of blood by PCR test is more usual. Although blood sample is more common for the molecular detection of human brucellosis, serum specimen is more popular and has priority over blood[51,52]. Several genus-specific multiplex PCR systems are developed based on primer pairs that target IS*711*, IS*650*, 16SRNA, *BCPS31*, and *omp2a* sequences. PCR can also be used for assessing the treatment efficacy, species differentiation, and biotyping of isolates[22,45,46]. The results of Alikhani *et al*.’s[53] study on the comparison of blood culture BACTECH system and whole blood and serum PCR method indicated that PCR can be considered as a sensitive and specific method for the diagnosis of human brucellosis. Moreover, in a study performed by Hajia *et al*.[54], SAT, Coombs Wright test, 2-mercaptoethanol test (2ME), ELISA (IgG and IgM), and PCR method have been compared using serum samples. Among the applied methods of diagnosis, the SAT displayed the lowest positivity rate and ELISA test had the highest efficiency. Also, the sensitivity of the PCR method was lower in comparison to ELISA.

### Treatment

Despite the application of WHO’s antibiotic regimen recommendation (1986), which consists of doxycycline 100 mg orally twice a day for 6 weeks plus oral rifampicin 600 to 900 mg daily for 6 weeks or streptomycin 1 g intramuscularly daily for 2-3 weeks, the rate of brucellosis treatment failure and relapse has been increased between 5-15% cases. The choice therapeutic regimen for the uncomplicated brucellosis consists of streptomycin for 2 to 3 weeks plus doxycycline for 8 weeks or gentamicin for 5-7 days plus doxycycline for 8 weeks[55]. The second-line agents such as quinolones or trimethoprim-sulfamethoxazole can be administered for patients with treatment failure or repeated relapses. For patients with a complicated disease, treatment intervention requires a careful evaluation of the patient and a thorough therapeutic plan. Patients with spondylitis should possibly receive a quinolone in the initial regimen, for a protracted period[56]. According to Kassiri *et al*.[28], the treatment regimen of doxycycline plus rifampin was used in 60.4% of brucellosis cases. However, Haddadi *et al*.’s[57] study in Tehran showed that the combination of cotrimoxazole and doxycycline was more effective in disease control.

### Control and prevention

#### Control and eradication

In 1998, WHO suggested general strategies as well as the Mediterranean Zoonoses Control Program for the eradication of animal brucellosis. The strategies and program included (1) prevention of disease extension among animals and monitoring brucellosis-free herds and regions, (2) identification of infected animals using diagnostic tests and their elimination by slaughter programs to generate brucellosis-free herds and zones, and (3) applying vast vaccination programs to decrease the disease prevalence. However, pasteurization of dairy products is considered as a significant safety method in endemic areas. Consumption of unpasteurized milk and dairy products and also raw or undercooked animal products (including bone marrow) should be avoided. Occupational exposure to *Brucella* can be prevented by good hygiene and using protective clothing/equipment. The use of safety measures are essential to prevent skin contamination, inhalation, or accidental ingestion of organisms while assisting at the birth, carrying out a necropsy, or butchering an animal. Moreover, handling an aborted fetus or its membranes and fluids requires especial precaution[4,19,35,39]. As described previously, consumption of unpasteurized milk and dairy products, slaughtered meat, and direct contact with animals are the main risk factors of brucellosis in Iran. Thus, improved veterinary services and public health education may play an important role in the disease control[36]. According to Esmaeili[25], there are numbers of major problems for brucellosis control in Iran that include: (1) lack of a proper law for the punishment of violators in animal health field, (2) weaknesses in border quarantine system and animal trafficking from neighboring countries, (3) lack of rural and nomadic livestock identification system, (4) nomadic and semi-nomadic conditions of small ruminant husbandry that make the control of animal movement very difficult, (5) keeping sheep more than the immunity period of Rev.1 vaccine in some areas, and (6) making low payment to veterinarians who fight against the disease in operation teams.

#### Prevention

Live, attenuated vaccines

Since presently there is no safe and protective human vaccine against brucellosis, animal vaccination is a critical factor for the control and eradication of animal and human brucellosis. An ideal vaccine against *Brucella* should: (1) prevent *Brucella* infection in both genders, (2) not provoke disease in immunized animals, (3) prevent abortion, (4) confer long-term protection with only one dose, (5) not interfere with LPS-based serological tests, (6) be biologically stable and not present the risk of virulence reversion, (7) not be pathogenic to humans, and (8) not contaminate the derivatives of the vaccinated animals[58]. At present, animal vaccination against *Brucella* infection is usually performed by the administration of the live attenuated smooth *Brucella* strains, including *B. abortus* strain S19, *B. abortus* strain RB51, and *B. melitensis* strain Rev.1[22]. Although live, attenuated vaccines promote long-term protection, they have major disadvantages: (1) causing abortion in pregnant animals, (2) secreting in milk of vaccinated animals, (3) being pathogenic to humans, (4) interfering with the LPS-based diagnostic tests, and (5) being resistant to rimfampicin, the first antibiotic of choice to human brucellosis treatment[22,59].

Since 1960s, vaccination with *B. melitensis* strain Rev. 1 has been considered as the main strategy for the control of brucellosis in small ruminant in Iran. In 1963, early studies in the production of Rev.1 vaccine, as a domesticated biological vaccine, were started in Razi Vaccine and Serum Research Institute of Iran in cooperation with WHO. Since 1963, testing the vaccine efficacy in goats and sheep has proven that REV.1 vaccine is able to decrease the epidemic rate of the disease from 45% to 1.8%[25]. During 1983–2003, vaccination program was limited to young animals, and a test-and-slaughter campaign was conducted in adult sheep and goats using Rose Bengal, SAT, and 2ME tests. From 2003, control program was based on the mass vaccination of lambs and kids at the age of 4–7 months, using full doses (1–3*×*10^9^ colony-forming units [CFU]) of Rev.1 vaccine, and also based on the immunization of the adult female animals with the reduced doses of the vaccine (0.5–2*×*10^6^ CFU). Additionally, upon performing other control programs, including public education, promotion of sanitary husbandry practices, and microbiological assessment of herds with abortion outbreaks, the number of annual new human cases was reduced from 39 in 2005-2006 to 15.9 in 2010-2011 per 100,000 population[17]. The first vaccination program was performed for cattle in 1949. Because of the high prevalence of bovine abortion due to *B*. *abortus*, vaccination of adult cows and 3-8-month-old calves has been started using S19 vaccine since 1967[60,61]. Adult vaccination using S19 vaccine was replaced by K45/20A in 1972, whereas vaccination with K45/20A was discontinued in 1980. Since 1988, all female cattle between 3-6 months of age have been vaccinated with S19, and hygiene education programs have been implemented for farmers. In 2007, S19 vaccine was removed from the brucellosis control program, and since then all cattle were immunized with RB51[60,61]. In order to control the bovine brucellosis in dairy cow farms, the Iranian Veterinary Organization has recently set up a control program using a mass vaccination with RB51 and also test-and-slaughter and quarantine, as eradication measures. Subcutaneous vaccination of calves with 1-3.4×10^10^ CFU of RB51 and the reduced dose of 1-3.4×10^9^ CFU for adult animals are officially recommended[62]. However, in 2007 in Iran, cases of abortion in dairy cows were reported by Sharifi *et al*.[62] following vaccination with strain RB51[62]. Pishva and Salehi[63] also reported the first isolation of *B. melitensis* vaccine strain REV.1 in cattle in Iran. The authors hypothesized that in traditional farms where cattle and sheep are kept in the same place, ewe vaccination (Rev.1) can be a source of *Brucella* infection and abortion in cattle.

Subunit vaccines

In the recent decades, subunit vaccines are becoming promising vaccine candidates against *Brucella* infection due to their safety profile. The major advantages of subunit vaccines over live vaccines is that subunit vaccines eliminate safety concerns associated with, they are less biohazardous, well defined, avirulent, noninfectious, and nonviable.

However, subunit vaccines cannot replicate the immunogenicity of live vaccines and thus, they are not as effective as live attenuated vaccines. In order to develop a new effective *Brucella* vaccine, selection of an immunogen with potential to induce an adequate immune responses (biased towards a Th1) and confer the high level of protection is essential[4,58]. Recently, immunoproteomics approaches considerably facilitated the identification of many immunogenic proteins in *Brucella*. Many studies have been performed to evaluate the efficacy of these immunogens. However, only a few immunogens have demonstrated significant protective efficacy, *in vivo*. In Iran, a number of studies have focused on evaluating the vaccine potency of the most appropriate *Brucella* immunogens, as univalent or multivalent recombinant protein, DNA, or DNA priming/protein boosting vaccine regimens (Table 3). The first world report on the evaluation of the protective efficacy of the 36 kDa outer membrane protein 2b (Omp2b) antigen and its truncated form has been performed by Golshani *et al*.[64,65]. Using immunoinformatics algorithms, mapping potential T- and B-cell epitopes are promising approaches to design new vaccine candidates. Furthermore, the numbers of immunogens have been analyzed using bioinformatics tools to design new vaccine targets based on epitope mapping [66-72].

**Table 3:** Subunit vaccine regimens and protective efficacies

Vaccine formula	Properties	Adjuvants	Immunization dose/route	Challenge stain/dose	Humoral immune response	Cellular immune response	Protection level (log10)	Ref.
1- rTOmp2b 2- pcDNA3.1-TOmp2b 3- pcDNA3.1-TOmp2b priming/ rTOmp2b boosting	Truncated 36 kDa Omp	CpG ODN 1826+Montanide ISA 70VG	rProtein: 30 µg/s.c. plasmid: 50 µg/s.c.	*B. melitensis* 16M/2×10^4^, *B. abortus* 544/ 4×10^4^	IgG2a↑, IgG1↓	IFN-γ↑, IL-10↓, IL-4↓	1- 0.71, 0.73 2- 0.48, 0.58 3- 0.88, 1.11	64
1- rSOmp2b 2- pcDNA3.1-SOmp2b 3- pcDNA3.1-SOmp2b priming/ rSOmp2b boosting	36 kDa Omp lacking the signal peptide	CpG ODN 1826+Montanide ISA 70VG	rProtein: 40 µg/s.c. plasmid: 50 µg/s.c.	*B. melitensis* 16M/2×10^4^, *B. abortus* 544/ 4×10^4^	IgG2a↑, IgG1↓	IFN-γ↑, IL-10↓, IL-4↓	1- 0.64, 0.81 2- 0.55, 0.75 3- 0.98, 1	65
PcDNA3.1- Omp31-eae	31kDa Omp, Omp intimin from *E. coli*	-	100 µg/i.m.	*B. melitensis* 16M, *E. coli* /10^4^ CFU	IgG2a↑, IgG1↓	IFN-γ↑, IL-10↓	NM	73
1- rL7/L12, 2- rTOmp31, 3- rL7/L12-TOmp31	ribosomal protein, truncated 31 kDa Omp, fusion protein	CpG ODN 1826+Montanide ISA 50V	15 µg, 15 µg, 30 µg/s.c.	*melitensis* 16M/2×10^4^, *B. abortus* 544/ 4×10^4^	IgG2a↑, IgG1↓	IFN-γ↑, IL-10↓	1- 1.3, 1.01 2- 1.16, 1.5 3- 1.13, 1.25	74
1- pcDNA3.1-L7/L12-TOmp31, 2- pcDNA3.1-L7/L12-TOmp31 priming/ rL7/L12-TOmp31 boosting	Fusion of ribosomal protein and truncated 31 kDa Omp	CpG ODN 1826+Montanide ISA 50V	rprotein: 30 µg/s.c. plasmid: 50 µg/s.c.	*B. melitensis* 16M/2×10^4^, *B. abortus* 544/ 4×10^4^	IgG2a↑, IgG1↓	IFN-γ↑, IL-10↓	1- 0.9, 1.1 2- 1.95, 1.7	75
rUrease	Enzyme	CFA/IFA	20, 30/ i.p., s.c.	*B. melitensis* 16M, *B. abortus* 544, *B. suis* 1330/2×10^7^ CFU	IgG1↑, IgG2a↑	IFN-γ↑, IL-10↑, IL-4↓	1.88, 2.21, NM	76
rOmp31+rTF	31 kDa Omp + Trigger factor	CFA/IFA	30 µg/i.p.	*B. melitensis* 16M/10^4^ CFU	IgG1↑, IgG2a↓	IFN-γ↑, IL-10↑, IL-4↓	2.27	77
1- rDnaK, 2- rTF, 3-rOmp31 4-rDnaK+rTF 5-rDnaK+rOmp31	Molecular chaperon, Trigger factor and 31kDa Omp	CFA/IFA	30 µg of each/i.p.	*B. melitensis* 16M/10^4^ CFU	IgG2a↑, IgG1↓	IFN-γ↑, IL-10↓	1- 1.63 2- 2.2 3- 1.66 4- 1.84 5- 1.88	78
rHspA	Heat shock protein	CFA/IFA	30 µg/i.p.	*B. melitensis* 16M/10^4^ CFU	IgG1↑, IgG2a↓	IFN-γ↑, IL-10↑, IL-4↑	1.49	79
rOmp19	19 kDa Omp	CFA	20 µg/s.c.	NM	Polyclonol Antisera↑	NM	NM	80
LPS	lipopolysaccharide	GBMOMV	10 µg/s.c.	NM	Total IgG↑	NM	NM	81
pcDNA3.1-Omp31	31kDa Omp	-	100 µg/i.m.	*B. melitensis* 16M/10^4^ CFU	IgG2a↑, IgG1↓	IFN-γ↑, IL-10↓	2.16	82
rHAS-L7/L12	Human Serum Albumin, ribosomal protein	-	10 µg/i.p.	*B. abortus* 544/5×10^5^ CFU	IgG1↑, IgG2a↓	NM	1.4	83

↑ shows induced production and ↓ indicates reduced production. NM, not mentioned; CFA, complete Freund’s adjuvant; IFA, incomplete Freund’s adjuvant; Ref. reference

All parts of Iran are endemic for brucellosis, and it causes high economic loss due to livestock abortion and has serious public health consequences. Brucellosis has been an occupational risk for people having contact with infected animals, and non-occupational source of the disease includes consumption of fresh and unpasteurized dairy products. The major problems for the control of brucellosis in Iran can be listed as following:

- Lack of public knowledge about brucellosis, especially in rural areas
- Public habit for consumption of raw milk and unpasteurized dairy products
- Lack of proper eradication program for infected animals
- Limitations of the commercially available animal vaccines
- Lack of the protective and safe human vaccine
- Keeping vaccinated livestock more than the protection period of the REV.1 vaccine
- Lack of proper border quarantine system and infected animal trafficking from neighboring countries
- Use of traditional livestock husbandry system in many rural areas
- Lack of livestock identification system in rural and nomadic areas causing the lack of information about animals’ immunization history
- Construction of animal barn near human house
- Lack of proper cooperation between farmers/livestock producers with veterinarians and Iran veterinary organization

With regard to these facts, educating farmers and people living in the endemic areas, routine screening of domestic livestock, inhibiting animal trafficking from infected neighboring countries, eliminating infected animals, and setting up vast vaccination programs can decrease the risk of both animal and human infection. Vast immunization of livestock is the most preventive program in the endemic countries like Iran; however, due to the major limitations of the current commercial available vaccines and the lack of the human vaccine, finding a new protective and safe vaccine target seems to be essential.

## References

[1] Pappas G (2010). The changing *Brucella* ecology:novel reservoirs, new threats. International journal of antimicrobial agent.

[2] Seleem MN, Boyle SM, Sriranganthan N (2010). Brucellosis:A re-emerging zoonosis. Veterinary microbiology.

[3] Vassallo DJ (1992). The corps disease:Brucellosis and its historical association with the royal army medical corps. Journal of the royal army medical corps.

[4] Perkins SD, Smither SJ, Atkins HS (2010). Towards a *Brucella* vaccine for humans. FEMS microbiology reviews.

[5] Zowghi E, Ebadi A, Yarahmadi M (2008). Isolation and identification of *Brucella* organisms in Iran. Iranian journal of clinical infectious diseases.

[6] Del-Vecchio VG, Kapatral V, Redkar RJ, Patra G, Mujer C, Los T, Ivanova N, Anderson I, Bhattacharyya A, Lykidis A, Reznik G, Jablonski L, Larsen N, Ds’Souza M, Bernal A, Mazur M, Goltsman E, Selkov E, Elzer PH, Hagius S, O’Callaghan D, Letesson JJ, Haselkorn R, Kyrpides N, Overbeek R (2002). The genome sequence of the facultative intracellular pathogen *Brucella melitensis*. PNAS.

[7] Purcell B, David L, Hoover DL, Friedlander AM, Brucellosis, Martha K, Lenhart MK, Colonel MC (2007). Medical aspects of biological warfare.

[8] Franco MP, Mulder M, Gilman RH, Smits HL (2007). Human brucellosis. Lancet infectious diseases.

[9] Tiller RV, Gee JE, Frace MA, Taylor TK, Setubal JC, Hoffmaster AR, De BK (2010). Characterization of novel *Brucella* strains originating from wild native rodent species in North Queensland, Australia. Applied environmental microbiology.

[10] Gwida M, Dahouk SA, Melzer F, Rösler U, Neubauer H, Tomaso H (2010). Brucellosis–Regionally emerging zoonotic disease?. Croatian medical journal.

[11] He Y (2012). Analyses of *Brucella* pathogenesis, host immunity, and vaccine targets using systems biology and bioinformatics. Frontiers cellular and infection microbiology.

[12] Young E, Gillespie S.H, Hawkey P.M (2006). *Brucella* spp. Principles and practice of clinical bacteriology.

[13] Delpy IP, Kaveh M (1945). The occurrence of brucellosis in Iran. The isolation of the causative agent of contagious abortion in the cattle. Rev de la face de med bet de the.

[14] Esmaeili H, Dehghani M (2012). Small ruminant brucellosis vaccination in Iran. Iranian journal of immunology.

[15] Refai M (2002). Incidence and control of brucellosis in the Near East region. Veterinary microbiology.

[16] Behroozikhah AM, Keyvanfar H, Feizabadi MM, Tabatabayi AH, Alamian S (2005). Differentiation of Iranian strains of *Brucella spp* by random amplification of polymorphic DNA. Archive of Razi institute.

[17] Behroozikhah AM, Bagheri Nejad R, KarimAmiri, Bahonar AR (2012). Identification at Biovar level of *Brucella* isolates causing abortion in small ruminants of Iran. Journal of Pathogens.

[18] Zowghi E, Ebadi A (1982). Typing of *Brucella* strains isolated in Iran. Archive of institute Razi.

[19] The Center for Food Security and Public Health (CFSPH) Brucellosis. Iwoa.

[20] Fallah Rostami F, Borzoueisileh S, Ebrahimpour S (2016). An overview of brucellosis epidemic in Iran. Crescent journal medical and biological sciences.

[21] Yang X, Skyberg JA, Cao L, Clapp B, Thornburg T, Pascual DW (2013). Progress in *Brucella* vaccine development. Frontiers in Biology.

[22] Corbel MJ (2006). Brucellosis in humans and animals.

[23] Von Bargen K, Gorvel JP, Salcedo SP (2012). Internal affairs:investigating the *Brucella* intracellular lifestyle. FEMS Microbiology reviews.

[24] Zeinali M, Shirzadi MR, Haj rasuliha H (2012). National Guideline for Brucellosis Control.

[25] Esmaeili H (2014). Brucellosis in Islamic republic of Iran. Journal of medical bacteriology.

[26] Mostafavi E, Asmand M (2012). Trend of brucellosis in Iran from 1991 to 2008. Iran journal of epidemiology.

[27] Rostami H, Mehrabi Tavana A, Tavakoli HR, Tutunchian M (2015). Prevalence study of brucellosis in Iranian military forces during 2001-2009. Journal of health policy and sustainable health.

[28] Kassiri H, Amani H, Lotfi M (2013). Epidemiological, laboratory, diagnostic and public health aspects of human brucellosis in Western Iran. Asian Pacific journal of tropical biomedicine.

[29] Khazaei S, Shojaeian M, Zamani R, Mansori K, Mohammadian-Hafshejani A, Rezaeian-Langroodi R, Ayubi E, Khazaei Z (2016). Epidemiology and risk factors of childhood brucellosis in West of Iran. International journal of pediatrics.

[30] Bokaie S, Sharifi L, Alizadeh H (2008). Epidemiological survey of brucellosis in human and animals in Birjand, East of Iran. Journal of animal veterinary advances.

[31] Zeinalian Dastjerdi M, Fadaei Nobari R, Ramazanpour J (2012). Epidemiological features of human brucellosis in central Iran 2006-2011. Public health.

[32] Azizi F, Hatami H, Janghorbani M (2010). Epidemiology and control of common diseases in Iran.

[33] Sofian M, Aghakhani A, Velayati AA, Banifazl M, Eslamifar A, Ramezani A (2008). Risk factors for human brucellosis in Iran:a case-control study. International journal of infectious diseases.

[34] Moosazadeh M, Abedi G, Kheradmand M, Safiri S, Nikaeen R (2016). Seasonal pattern of brucellosis in Iran:A systematic review and meta-analysis. Iranian journal of health sciences.

[35] Avila-Calderón ED, Lopez-Merino A, Sriranganathan N, Boyle SM, Contreras-Rodríguez A (2013). A history of the development of *Brucella* vaccines. Biomedical medical international.

[36] Alavi SM, Mugahi S, Nashibi R, Saeid Gharkholu S (2014). Brucellosis risk factors in the southwestern province of Khuzestan, Iran. International journal of enteric pathogens.

[37] Beheshti S, Rezaian GR, Azad F, Faghiri Z, Taheri F (2010). Seroprevalence of brucellosis and risk factors related to high risk occupational groups in Kazeroon, South of Iran. International journal of occupational environment medicine.

[38] Akbarmehr J (2011). The prevalence of *Brucella abortus* and *Brucella melitensis* in local cheese produced in Sarab city, Iran and its public health implication. African journal of microbiol research.

[39] Bahador A, Mansoori N, Esmaeili D, Amini Sabri R (2012). Brucellosis:Prevalence and retrospective evaluation of risk factors in western cities of Tehran province, Iran. Journal of bacteriology research.

[40] Hasanjani Roushan MR, Mohrez M, Smailnejad Gangi SM, Soleimani Amiri MJ, Hajiahmadi M (2004). Epidemiological features and clinical manifestations in 469 adult patients with brucellosis in Babol, Northern Iran. Epidemiology and infection.

[41] Xavier MN, Paixão1 TA, den Hartigh AB, Tsolis RM, Santos RL (2010). Pathogenesis of *Brucella* spp. The open veterinary science journal.

[42] Galińska EM, Zagórski J (2013). Brucellosis in humans- etiology, diagnostics, clinical forms. Annals of agricultural and environmental medicine.

[43] Zamani A, Kooraki S, Adabi Mohazab R, Zamani N, Matloob R, Hayatbakhsh MR, Raeeskaramia SR (2011). Epidemiological and clinical features of *Brucella* arthritis in 24 children. Annals of Saudi medicine.

[44] Azizi F, Katchoui A (1996). *Brucella* infection of the thyroid gland. Thyroid.

[45] Dahouk S, Nöckler K (2011). Implications of laboratory diagnosis on brucellosis therapy. Expert review of anti-infective therapy.

[46] Coelho AC, Garcia Diez J (2015). Biological risks and laboratory-acquired infections:A reality that cannot be ignored in health biotechnology. Frontiers in bioengineering and biotechnology.

[47] Baddour MM (2012). Diagnosis of brucellosis in humans:a review. Journal of veterinary advances.

[48] Christopher S, Umapathy BL, Ravikumar KL (2010). Brucellosis:review on the recent trends in pathogenicity and laboratory diagnosis. Journal of laboratory physicians.

[49] Corbel MJ (1997). Brucellosis:an overview. Emerging infectious diseases journal.

[50] Mantur BG, Mangalgi SS (2004). Evaluation of conventional Castaneda and lysis centrifugation blood culture techniques for diagnosis of human brucellosis. Journal of clinical microbiology.

[51] Yu WL, Nielsen K (2010). Review of detection of *Brucella* spp. by polymerase chain reaction. Croatian medical journal.

[52] Zerva L, Bourantas K, Mitka S, Kansouzidou A, Legakis NJ (2001). Serum is the preferred clinical specimen for diagnosis of human brucellosis by PCR. Journal of clinical microbiology.

[53] Alikhani MY, Hashemi SH, Naseri Z, Farajnia S, Peeri-Dogaheh H (2012). Diagnosis of human brucellosis by blood culture (BACTEC) and PCR method via whole blood and serum. Jundishapur journal of microbiology.

[54] Hajia M, Fallah F, Angoti G, Karimi A, Rahbar M, Gachkar L, Mokhtari B, Sanaei A, Rastegar Lari A (2013). Comparison of methods for diagnosing brucellosis. Science.

[55] Ranjbar M (2015). Updates on Brucellosis:Treatment of Brucellosis.

[56] Alavi SM, Alavi L (2013). Treatment of brucellosis:a systematic review of studies in recent twenty years. Caspian journal of international medicine.

[57] Haddadi A, Rasoulinejad M, Afhami SH, Mohrez M (2006). Epidemiological, clinical and para clinical aspects of brucellosis in Imam Khomeini and Sina hospitals of Tehran (1998-2009). Journal of Kermanshah university of medical sciences.

[58] Oliveira SC, Macedo GC, Almeida LA, Oliveira FS, Oñate A, Cassataro J, Giambartolomei GH (2010). Recent advances in understanding immunity against brucellosis:application for vaccine development. The open veterinary science journal.

[59] Cassataro J, Estein SM, Pasquevich KA, Velikovsky CA, de la Barrera S, Bowden R, Fossati CA, Giambartolomei GH (2005). Vaccination with the recombinant *Brucella* outer membrane protein 31 or a derived 27-amino-acid synthetic peptide elicits a CD4+T helper 1 response that protects against *Brucella*
*melitensis* infection. Infection and immunity.

[60] Esmaeili H, Tajik P, Ekhtiyarzadeh H, Bolourchi M, Hamedi M, Khalaj M, Amiri K (2012). Control and eradication program forbovine brucellosis in Iran:An epidemiological survey. Journal of veterinary research.

[61] Esmaeili H, Salehi B (2012). Bovine brucellosis vaccination in IRAN. Iranian journal of immunology.

[62] Yazdi HS, Kafi M, Haghkhah M, Tamadon A, Behroozikhah AM, Ghane M (2009). Abortions in pregnant dairy cows after vaccination with *Brucella abortus* strain RB51. Veterinary record.

[63] Pishva EM, Salehi M (2008). First report of isolation of *Brucella melitensis* vaccine strain rev.1 as a source of cattle infection in Iran. Journal of Sciences Islamic Republic of Iran.

[64] Golshani M, Rafati S, Nejati-Moheimani M, Ghasemian M, Bouzari S (2016). Comparison of potential protection conferred by three immunization strategies (protein/protein, DNA/DNA, and DNA/protein) against *Brucella* infection using Omp2b in BALB/c Mice. Veterinary microbiology.

[65] Golshani M, Rafati S, Nejati-Moheimani M, Pourabdi S,d, Arsang A, Bouzari S (2017). Protein/protein, DNA/DNA and DNA/protein based vaccination strategies using truncated Omp2b against *Brucella* infection in BALB/c Mice. International journal of medical microbiology.

[66] Golshani M, Rafati S, Jahanian-Najafabadi S, Nejati-Moheimani M, Siadat SD, Shahcheraghi F, Bouzari S (2015). *In silico* design, cloning and high level expression of L7/L12-TOmp31 fusion protein of *Brucella* antigens. Research in pharmaceutical sciences.

[67] Golshani M, Vaeznia N, Sahmani M, Bouzari S (2016). *In silico* analysis of *Brucella abortus* Omp2b and *in vitro* expression of SOmp2b. Clinical and experimental vaccine research.

[68] Golshani M, Ghasemian M, Gheibi N, Bouzari S (2017). *In silico* design, and *in vitro* expression of a fusion protein encoding *Brucella abortus* L7/L12 and SOmp2b antigens. Advanced biomedical journal.

[69] Ghasemi A, Ranjbar R, Amani J (2014). *In silico* analysis of chimeric TF, Omp31 and BP26 fragments of *Brucella melitensis* for development of a multi subunit vaccine candidate. Iranian journal of basic medical sciences.

[70] Sekhavati MH, Majidzadeh Heravi M, Tahmoorespur M, Yousefi S, Abbassi-Daloii T, Akbari R (2015). Cloning, molecular analysis and epitopics prediction of a new chaperone GroEL *Brucella melitensis* antigen. Iranian journal of basic medical sciences.

[71] Yousefi S, Tahmoorespur M, Sekhavati MH (2015). B and T-Cell epitope prediction of the OMP25 antigen for developing *Brucella melitensis* vaccines for sheep. Iranian journal of applied animal sciences.

[72] Tahmoorespur M, Sekhavati MH, Yousefi S, Abbassi-Daloii T, Azghandi M, Akbari R (2016). *In silico* analysis of Omp25 and BLS *Brucella melitensis* antigens for designing subunit vaccine. Archives Razi institute.

[73] Ranjbar R, Sharifimoghadam S, Saeidi E, Jonaidi N, Sheikhshahrokh A (2016). Induction of protective immune responses in mice by double DNA vaccine encoding of *Brucella melitensis* Omp31 and *Escherichia coli* Eae genes. Tropical journal of pharmaceutical research.

[74] Golshani M, Rafati S, Gholami E, Siadat SD, Oloomi M, Bouzari S (2015). Vaccination with recombinant L7/L12-Truncated Omp31 protein induces protection against *Brucella* infection in BALB/c mice. Molecular immunology.

[75] Golshani M, Rafati S, Siadat SD, Nejati-Moheimani M, Shahcheraghi F, Arsang A, Bouzari S (2015). Improved immunogenicity and protective efficacy of a divalent DNA vaccine encoding *Brucella* L7/L12-truncated Omp31 fusion proteinby a DNA priming and protein boosting regimen. Molecular immunology.

[76] Abkar M, Amani J, Sahebghadam Lotfi A, Nikbakht Brujeni G, Alamian S, Kamali M (2015). Subcutaneous immunization with a novel immunogenic candidate (urease) confers protection against *Brucella abortus* and *Brucella melitensis* infections. Acta pathologica, microbiologica, et immunologica Scandinavica.

[77] Ghasemi A, Jeddi-Tehrani M, Mautner J, Salari MH, Zarnani AH (2015). Simultaneous immunization of mice with Omp31 and TF provides protection against *Brucella melitensis* infection. Vaccine.

[78] Ghasemi A, Jeddi-Tehrani M, Mautner J, Salari MH, Zarnani AH (2014). Immunization of mice with a novel recombinant molecular chaperonconfers protection against *Brucella melitensis* infection. Vaccine.

[79] Ghasemi A, Zarnani AH, Ghoodjani A, Rezania S, Salari MH, Jeddi-Tehrani M (2014). Identification of a new immunogenic candidate conferring protection against *Brucella melitensis* infection in mice. Molecular immunology.

[80] Farahi F, Asli E, Mohabati Mobarez A, Khoramabadi N, Bakhtiari A (2012). Recombinant *Brucella abortus* outer membrane protein 19 (rOmp19) significantly stimulates splenic lymphocytes of immunized BALB/c mice. African J Microbiol Res.

[81] Sharifat Salmani A, Siadat SD, Norouzian D, Izadi Mobarakeh J, Kheirandish M, Zangeneh M, Aghasadeghi MR, Nejati M, Hedayati MH, Arfa Moshiri A, Sadat SM (2009). Outer membrane vesicle of *Neisseria meningitidis* serogroup B as an adjuvant to induce specific antibody response against the lipopolysaccharide of *Brucella abortus* S99. Annals microbiol.

[82] Doosti A, Ghasemi-Dehkordi P, Javadi GHR, Sardari S, Shokrgozar MA (2009). DNA vaccine encoding the Omp31 gene of *Brucella melitensis* induces protective immunity in BALB/c mice. Research journal of biological sciences.

[83] Pakzad I, Rezaee A, Rasaee MJ, Tabbaraee B, Delpisheh A (2009). Immunogencity of HSA-L7/L12 (*Brucella abortus* ribosomal protein) in an animal model. Iranian journal of immunology.

